# Static Magnetic Field (SMF) as a Regulator of Stem Cell Fate – New Perspectives in Regenerative Medicine Arising from an Underestimated Tool

**DOI:** 10.1007/s12015-018-9847-4

**Published:** 2018-09-17

**Authors:** Krzysztof Marycz, K. Kornicka, M. Röcken

**Affiliations:** 1 Department of Experimental Biology, Wroclaw University of Environmental and Life Sciences, Norwida 27B, Wrocław, Poland; 2 Faculty of Veterinary Medicine, Equine Clinic - Equine Surgery, Justus-Liebig-University, 35392 Gießen, Germany

**Keywords:** Stem cells, Magnetic field, Differentiation, Signalling pathways

## Abstract

Tissue engineering and stem cell-based therapies are one of the most rapidly developing fields in medical sciences. Therefore, much attention has been paid to the development of new drug-delivery systems characterized by low cytotoxicity, high efficiency and controlled release. One of the possible strategies to achieve these goals is the application of magnetic field and/or magnetic nanoparticles, which have been shown to exert a wide range of effects on cellular metabolism. Static magnetic field (SMF) has been commonly used in medicine as a tool to increase wound healing, bone regeneration and as a component of magnetic resonance technique. However, recent data shed light on deeper mechanism of SMF action on physiological properties of different cell populations, including stem cells. In the present review, we focused on SMF effects on stem cell biology and its possible application as a tool for controlled drug delivery. We also highlighted the perspectives, in which SMF can be used in future therapies in tissue engineering due to its easy application and a wide range of possible effects on cells and organisms.

## Introduction

In the age of industrial and technological development, there is a strong demand from societies for the development of physics-based medicine, which may offer new treatment options, especially for patients suffering from chronic diseases. In the last 20 years, several research groups from different parts of the world have been developing and investigating physics-related devices, including systems based on static magnetic field (SMF) for physics-based medicine. The most common use of SMF can be found in magnetic resonance imaging (MRI). The interaction of livings cells, organs or experimental animals with a magnetic field has inspired a broad spectrum of research groups from different fields, including cell and molecular biology, medicine, nanobiotechonology and physics. However, it is still necessary to better understand the action of SMF at the molecular level, with special emphasis on its effect on cell communication, behavior and secretory activity. There is an increasing number of clinically approved medical devices, including neodymium magnets, magnetic nanoparticles or magnetic biomaterials that are introduced into medical and veterinary markets; they are proposed to be applied as an additional and supplementing medication or rehabilitation treatment methods. It seems that understanding SMF physics will allow to optimize, validate and evaluate the safety and efficacy of magneto therapy in selected treatments in both humans and animals.

There are contradicting data regarding the beneficial effect of SMF on patients’ health, therefore, it is strongly required to explore the knowledge in this field, especially that SMF may be a useful system for the controlled release of active agents, including drugs, growth factors or miRNA. Moreover, SMF among many other factors like endurance exercise [1–3], bioactive compounds [4] may contributes to mobilization of circulating progenitor cells in peripheral blood as well as in bone marrow. However, the biological effect of SMF seems to be poorly discussed, particularly in the context of stem cell physiology as well as regenerative medicine in both humans and animals. Therefore, we would like to focus in this review on selected aspects of regenerative medicine, stem cells as well as intercellular signaling in relation to static magnetic field, bearing in mind conflicting data that have been recently published.

## MF in Stem Cell Signaling and Differentiation

Static magnetic field (SMF) is a constant, non-changing vector field that describes the magnetic influence of electrical currents and magnetized materials on living and inanimate matter. SMF is classified as a weak (<1 mT), moderate (1mT to 1 T), strong (1 T to 5 T) and ultrastrong (>5 T) field. This classification, which has been accepted in the scientific community, was proposed to create clear SMF ranges to allow consistent research and clarification of its biological and therapeutic potential. Unlike other magnetic fields (including electromagnetic and non-ionizing), SMF is more convenient to apply in therapy, because only simple magnetic discs are used to generate it both in vitro and in vivo. Over the years, SMF has been widely applied in physiotherapy for the treatment of bone disorders, including osteoarthritis. However, recently, SMF has gained the attention of scientist working in the fields of stem cells and tissue engineering. Adult stem cells are continuously affected by multiple external stimuli, such as trophic factors, fluid shear stress and hydrostatic pressure. Both stem cell niches and internal stimuli affect stem cell behavior and differentiation potential [5, 6]. Moreover, previous studies have indicated that cells are able to communicate by sending and receiving electromagnetic cues [7, 8]. Thus, the application of SMF and its possible effects on stem cell fate pose an interesting perspective in the field of tissue engineering, in which these cells are applied to regenerate damaged tissues and organs. Interestingly, it has been noted that endogenous electrical potentials appear in wounded tissues and successively disappear during the regeneration process. For that reason, the application of SMF should depend on the stage of the healing. Nevertheless, the biological effects of SMF on stem cell populations still need to be fully elucidated. Mesenchymal stem cells (MSCs) are a population of adult stem cells that can be isolated from multiple tissues, including bone marrow, adipose tissue and dental pulp [9]. MSCs are well known for their immunomodulatory properties and multilineage differentiation potential, thus they have attracted interest as a useful tool for cell-based therapies [10–12]. Therefore, it is interesting to evaluate whether exposure to SMF affects MSC fate not only in vitro but also in vivo, especially that SMF effects on stem cell biology are still poorly understood.

SMF can affect stem cell fate decision in many ways. It has been shown that magnetic field can affect the concentration of ions within the cytoplasm, including Ca^2+^. A study performed by Koch et al. has demonstrated that extremely low frequency (ELF) magnetic fields, ranging from 27 to 37 mT, can regulate Ca^2+^ transport by interacting with Ca^2+^ channels in the cell membrane [13]. Electromagnetic field (50 Hz, 20 mT) exposures led in MCSs to the activation of Na+/K+ channels, resulting in an increase in Na+/K+ concentration [14]. Modulation of key ion distributions affects in consequence stem cell function, proliferation and differentiation. Increased levels of calcium ions in the cytoplasm may, in consequence, trigger changes in the actin microdomain and distribution, thereby affecting cell shape and geometry. SMF affects cell size, shape, membrane surface and distribution of cellular organelles by modulating Ca^2+^ concentration and distribution of actin filaments [15]. A similar phenomenon was observed in human ASCs exposed to SMF, where organelles were translocated to a specific pole [16], leading to the restoration of cell polarity. Nucleus and other organelles were concentrated in one of the poles. Cytoskeletal rearrangements affect different types of mechanoreceptors, including integrins. Cells use integrin receptors in order to adhere to the proteins forming the extracellular matrix and transduce mechanical cues in and out of the cell body. It has been shown that SMF can influence proliferation, migration, and adhesion of human vascular smooth muscle cells by inhibiting the clustering of integrin β1 [17]. In ASCs, 0.5 T SMF induced the expression of αV and β3 integrins, which mediate the shear stress-induced cell migration [18]. Furthermore, SMF exerted anti-apoptotic effects on ASCs, observed as decreased expression of p21, p53 and BAX. What is more, ASCs cultured in the presence of SMF were characterized by an increased proliferation rate in comparison to the cells cultured in standard conditions.

Stem cell fate is also tightly regulated by multiple signaling pathways, transcription factors and other molecular mechanisms [19–21]. In a recent study by Lew et al., it was shown that 0.4 T SMF significantly enhanced the proliferation of dental pulp stem cells by activating the p38 mitogen-activated protein kinase (MAPK) pathway [22]. Similar results were obtained by Wang et al., who found that p38 played a crucial role in the SMF cell response. The phosphoinositide 3-kinase/Akt (PI3K/Akt) pathway plays a crucial role in pluripotency and cell fate determination, regulating cell proliferation, survival and metabolism [23]. Accordingly, a study conducted by Marędziak et al. revealed that 0.5 T SMF enhanced the proliferation and viability of adipose-derived mesenchymal stem cells (ASCs) via the PI3K/Akt pathway [18]. Moreover, it reduced the expression of apoptosis-promoting genes, e.g., p53, p21 and BAX.

Recently, special attention has been paid to stem cell-derived microvesicles (MVs), which transport a wide range of cargo including proteins, organelles, RNA, miRNA and bioactive lipids [24, 25]. MVs are enriched in bioactive molecules, thus they play a pivotal role in many biological processes, including tissue regeneration. Therefore, increased secretion of stem cell-derived MVs caused by the application of SMF would be highly desirable in the context of effective therapy. Studies performed by Marędziak et al. [26, 27] demonstrated that 0.5 T SMF enhanced the synthesis of MVs enriched with VEGF and BMP-2 in equine ASCs. Similar results were obtained by Stratton et al. [28], however, they used pulsed MF to enhance the synthesis of MVs from monocytic leukemia cells. ASC-derived MVs contain multiple paracrine factors, which primarily contribute to their therapeutic potential observed in clinical trials. However, technical challenges limit the effective harvesting of MVs and their clinical application. What is more, concerns regarding the affordability of large-scale MV production still exist. Thus, the application of SMF in order to enhance secretion of ASC-derived MVs may overcome those issues, because it is a simple and non-expensive procedure that can be applied both in vitro and in vivo.

Positive effects of SMF application, e.g., cytokine and growth factor secretion, cell migration and proliferation have been observed in the range from 600 μT tp 9.4 T. However, it should be taken into account that those plausible effects of SMF are not only intensity- but also time- and cell type-dependent. For example, exposure to electromagnetic field (EMF) (15 Hz, 5 mT, by 21 days) increased the expression of collagen type II and glycosaminoglycan content in human mesenchymal stem cells (MSCs) [29]. A study by Kasten et al. [30] demonstrated that SMF stimulated the expression of Sox-9 and VEGF, while it reduced the expression of ALP and PPARγ in human bone marrow-derived mesenchymal stem cells (BMSCs). Another study performed by Marędziak et al. [27] demonstrated that 0.5 T SMF exposure resulted in increased proliferation of equine ASCs. In addition, it was demonstrated that the application of SMF to human ASCs resulted in the formation of osteo-nodules without the addition of osteogenesis-inducing medium [16]. Furthermore, these osteonodules were characterized by a high mineralization ratio comparable to osteoblastic cells, which indicated that SMF had strong pro-osteogenic properties. It was also shown that SMF induced osteoblast differentiation by enhancing the expression of osteogenesis master regulator genes, e.g., ALP, Col-I, OPN, OCL and BMP-2 [16]. On the other hand, adipogenic differentiation of human ASCs was alleviated by SMF, as Oil Red O staining revealed the formation of small lipid deposits in comparison to the control group. However, the data regarding stem cell differentiation under SMF are still limited, as previous studies have mainly focused on osteoblastic cells. The effects of SMF on stem cell fate and molecular mechanisms associated with this process still needs to be fully elucidated.

Data obtained from multiple experiments strongly support the idea of SMF application in order to stimulate tissue regeneration. Previous studies have confirmed that mechanical forces play a significant role in regulating MSC fate [31]. Actin and integrin distribution plays a key role in differentiation, because it directly affects stem cell fate. For example, MSCs with spread actin cytoskeleton will likely undergo osteogenic differentiation, while those with spherical shape and dispersed cytoskeleton will preferably differentiate into adipogenic and chondrogenic lineages. Therefore, MSC fate can be modulated by actin-integrin rearrangement mediated by the magnetic field. It was demonstrated that it modulated stem cells fate, enhanced wound healing and improved bone healing after fractures. SMF has been shown to exert different effects on stem cells, including enhanced secretion of MVs enriched in growth factors, which has great implications for future and novel strategies in regenerative medicine. For example, the release of VEGF and other growth factors can be triggered and tuned by the application of SMF.

## Static Magnetic Field (SMFs) – A Potential Therapeutic Tool in Human and Animal Regenerative Medicine

Recently, the potential biomedical application of SMF has been extensively studied in the context of its beneficial effect on health. Increasingly more is known about the action of SMF, based on the research using various cellular models and advanced molecular techniques, with special emphasis on its therapeutic effect. Magnetotherapy with a low-frequency magnetic field has been officially approved by US Food and Drug Administration (FDA) for orthopedic applications, in treating pain and edema in superficial soft tissues [32]. Moreover, SMF generated by neodymium magnets and ferrite magnets, which generate low static magnetic field is considered safe by the National Center of Complementary and Alternative Medicine (NCCAM), which in consequence encourages the scientific community for testing its clinical application. The beneficial clinical effect of SMF of different strength, including low, moderate and high has been demonstrated. Weak SMF, up to 70 μT, has been classified by Heisenberg as an elementary energy, on which the organism life is dependent and which has become an indispensable part of humans life, because it constantly surrounds us (2). It was demonstrated that the lack of the natural, weak SMF causes in humans insomnia, fatigue and depression, and increases the risk of osteoporosis [34]. As explained, SMF stimulates the movement of cellular ions, increases the use of oxygen by the cell and activates integrins, and thus it affects cell fate by modulating cellular metabolism, clonogenic potential, cell cycle, proliferation as well as apoptosis (SMF has been shown to have beneficial effects for pain management, peripheral nerve regeneration, inflammation, cutaneous microcirculation, blood flow and pressure and united fractures [35–37]. It was shown that SMF exerted its anti-inflammatory properties by enhancing the secretion of IL-10, while controlling the secretion of pro-inflammatory cytokines, such as IL-6, IL-8 or TNF-α [38]. However, the most frequent application of SMF takes place in the field of musculoskeletal system disorders in both animals and humans regenerative medicine. It was demonstrated that the exposure to moderate SMF enhanced cartilage as well as bone regeneration by improving extracellular matrix formation [39–42]. Although there are limited research data regarding weak SMF in the context of its beneficial clinical effect, it was shown that the application of low frequency magnetic field reduced the inflammation and degenerative changes in the course of joint osteoarthritis in human [34]. Moreover, it was demonstrated that SMF in the range from 50 to 180 mT reduced pain and improved the functional status in patients with rheumatoid arthritis. A similar clinical effect was observed in osteotomy dogs, which were exposed to 0.3 mT and exhibited improved radiographic healing of osteotomy sites [43]. Intermediate SMF, i.e., 64 mT affected motor activity during sleep, reduced pain and allowed to reduce the administration of NSAIDs [33]. In addition, it was shown that 2 mT magnetic field was successfully used in arthritis treatment [44]. In turn, moderate-intensity SMF was shown to promote new bone formation, prevent decreased mineral bone density as well as induce metabolic activity of human and rodents cartilage [42, 43, 45, 46] In addition, it was shown that moderate SMF had an anti-inflammatory effect and reduced edema [47]. Interestingly, Kotani and his colleagues [48] showed both in vitro and in vivo that the exposure to strong SMF (8 T) stimulated bone formation through increased matrix formation and osteoblast differentiation. In the latter study, it was shown for the first time that strong SMF improved ectopic bone formation in and around subcutaneously implanted bone morphogenetic protein (BMP) 2 by regulating orientation of osteoblast (MC3T3-E1 cells) growth. The authors concluded that the combination of SMF together with potent factors affecting bone regeneration might be a future perspective approach in the field of bone regenerative medicine. It should be emphasized that apart from the strict pro-regenerative effect of SMF on bone regeneration, additional benefits have also been observed; they include enhancing effect on neo-vascularization process, pain relief, antiedematous and anti-inflammatory effect.

Additionally, it is worth noting that in musculoskeletal injuries and post-surgical treatment, reduction of edema has become a major therapeutic agent in the acceleration of pain and stress relief, which enhances the healing processes.

## MF as a Tool for Delivery of Active and Regulatory Agents (miRNA/Drugs)

**Fig. 1:**
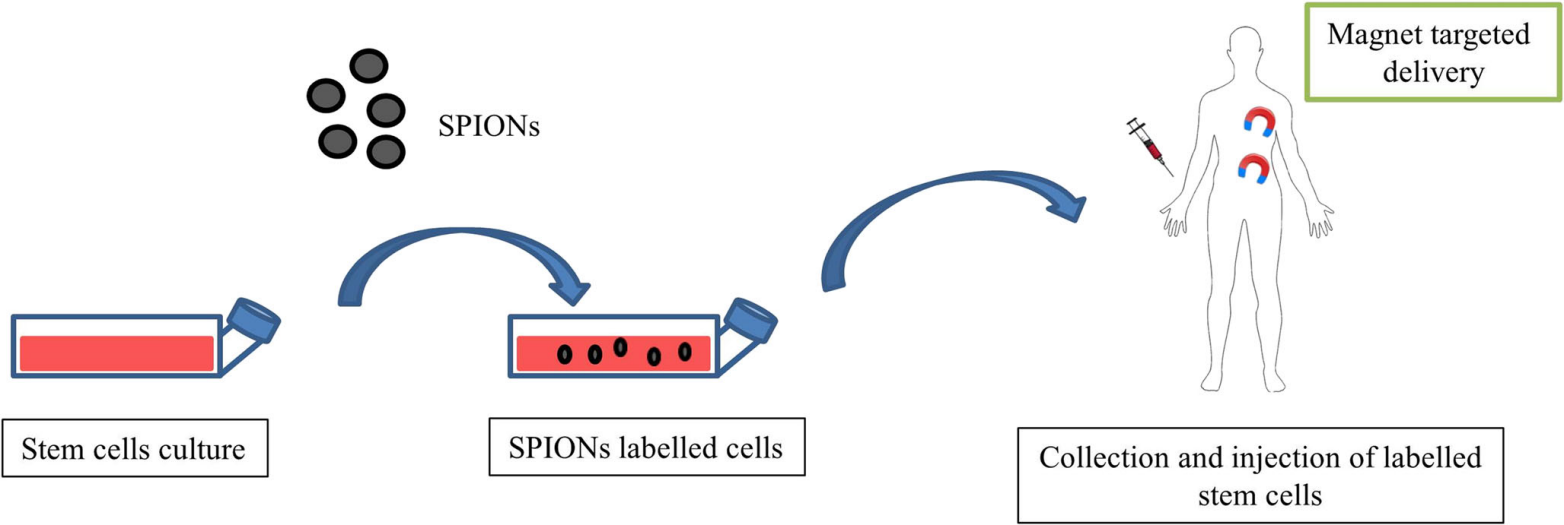
Application of magnetic targeted stem cell delivery to damaged organs

Over past years, magnetic field has emerged as a promising drug delivery system that provides controlled drug release. An important challenge in the treatment of cancer and many other diseases is to discover a technology enabling the controlled and targeted drug delivery and release to desirable cells, while sparing healthy ones. To address this issue, the most attention has been paid to magnetic nanoparticles (MNPs) that serve as delivery vehicles. They present a wide range of applications, including hyperthermia agents, magnetic guided vectors, drug carriers and imaging contrast probes [49–51]. More importantly, using SMF, these particles can direct selected drug delivery and enhance its local concentration in the affected tissue as well as release the drug “on demand”. The main clinical advantages of MNPs are small sizes, relatively easy preparation, good biocompatibility, efficient drug conjugation and superior magnetic responsiveness [52]. Thus far, MNPs based on iron oxides (IOs) (Fe3O4, Fe2O3) have been most broadly applied in medical research. They are characterized by excellent biocompability, low cytotoxicity and rapid response to an externally applied magnetic field, which makes them a potent tool in the advanced biomedicine applications. Superparamagnetic iron oxide (SPION) nanoparticles have been widely used to track the fate of transplanted cells in vivo [53]. However, a study performed by Ka-Wing et al. [54] demonstrated that SPION-labeled embryonic stem cells transplanted via direct intra-myocardial injection to the infarcted myocardium significantly improved heart function and enabled cellular tracking. On the other hand, SPION-labeled MSCs showed better migration and homing effects in vivo in mice with olfactory bulb damage [55]. A scheme depicting application of magnetic targeted stem cell delivery to damaged organs is shown on Fig. 1.

**Fig. 2:**
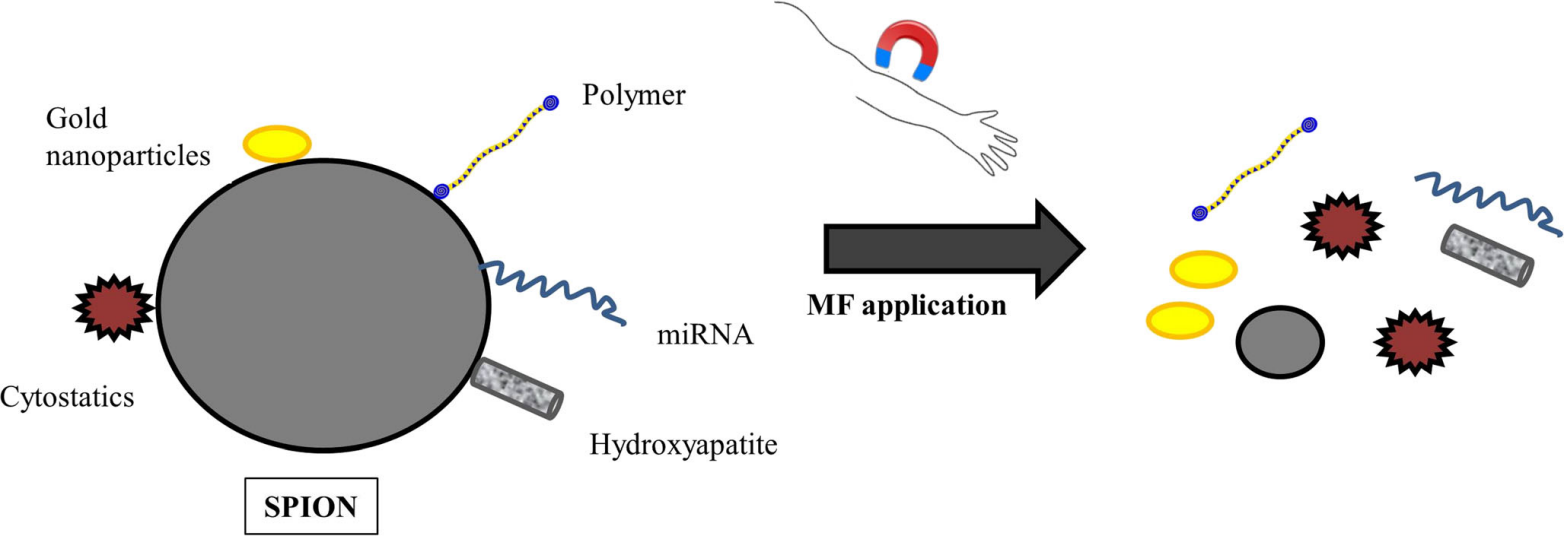
Controlled release of therapeutic agents via magnetic field application

Most recently, IOs have also been applied in tissue engineering as compounds for the production of innovative biomaterials. Salecan-g-poly(VA-co-HEA)/Fe3O4@Agarose hydrogels were demonstrated to release doxorubicin hydrochloride in the presence of magnetic field. On the other hand, IO nanoparticles were shown to be structurally stable in MSCs and promoted their osteogenic differentiation by upregulating long noncoding RNA, INZEB2. Huang et al. [56] have fabricated SPIONs co-coated with PEG and PEI polymers and further functionalized with folic acid (FA) in order to accomplish cancer specific targeting. Furthermore, anticancer drug, doxorubicin, was deposited on prepared nanoparticles. The application of magnetic field on DOX@FA-SPIONs on MCF-7 cells in vitro and on xenograft MCF-7 in nude mice with breast tumor in vivo significantly decreased tumor cell number and its growth. Another study showed that SPIONs conjugated to erlotinib released the drug intracellularly rather than into the bloodstream and precisely recognized and destroyed CL1–5-F4 cancer cells [57]. Among therapeutics that can be delivered using IOs, miRNA has emerged as a new potent biomolecule for use in regenerative medicine. These molecules work via base pairing with target RNA to negatively regulate its expression. Leder et al. [58] prepared silica-based micron-sized iron oxide-containing particles (sMPIO) that were able to target specific miRNA. Studies performed in vitro in primary hepatocytes revealed rapid particle uptake (4 h) followed by a significant depletion of the targeted microRNA Let7g (80%). Moreover, the up-regulation of target proteins, Cyclin D1, c-Myc, as well as specific proteome changes were noted. Nevertheless, SMF-controlled drug release has been mainly studied in cell lines. As regards stem cells, the data is elusive and further research needs to be conducted to characterize the target delivery of biomolecules to stem cell populations. One of the few available studies revealed that a magnetic non-viral vector fabricated with cationic polymer, polyethylenimine (PEI), bound to iron oxide magnetic nanoparticles (MNP), conjugated with miR-335, which regulated genes involved in the proliferation and differentiation of MSC. The obtained data revealed that complexes were characterized by a ~75% uptake efficiency and moderate cytotoxicity in MSCs. The delivery based on magnetic nanoparticles allows for successful and long-term transfection of hard-to-transfect miRNA into problematic cells, such as MSCs, thereby showing therapeutic potential in the regeneration of damaged tissues. In the future, IO nanoparticles may be conjugated with selected miRNAs and/or its inhibitors to alleviate the pathological state during different diseases with miRNA involvement. Controlled miRNA release will not only allow for a time-, but also dose-dependent molecule release by simple application of an external magnetic field. This approach may become beneficial in the treatment of bone injuries, osteoarthritis and arthritis. For example, selected anti-inflammatory miRNA may be released into the joint capsule during arthritis at selected time points by applying SMF to the knee area. Similar attempts can be made in the production of scaffolds for bone regeneration, as delivered miRNA may regulate the activity of cells involved in bone remodeling, e.g., osteoblasts and osteoclasts as well as endogenous stem cells to enhance their differentiation. Possibilities of magnetic field application in controlled release of therapeutic agents are shown at Fig. 2.

## Future Perspectives for Clinical Application of MF

Static magnetic field (SMF) is an increasingly recognized supplementary medicine tool, which can improve regenerative processes of the body by modulating the metabolism of individual cells. Different strength of SMF can be used to control stem cell differentiation in vitro before transplantation. SMF has been shown to affect osteogenic, chondrogenic and adipogenic cells, which in turn offers new therapeutic opportunities. The pre-treatment of stem cells using SMFs toll before their clinical application to obtain specific cell properties seems to be a logical consequence of the knowledge collected by different research groups worldwide. Moreover, based on the published data, it can be speculated that the application of different SMF strengths in stem cell cultures can induces the synthesis and secretion of specific extracellular microvesicles (ExMVs), which are an important part of intercellular signaling. The ability to control ExMV transfer between damaged tissues may provide an improvement in regenerative processes. It cannot be overlooked that SMF can be successfully used to control the delivery of active substances, drugs, growth factors or therapeutic miRNA. From that perspective, SMFs may offer underestimated opportunity to target specific tissues and/or organs by an accurate spatial and temporal delivery of therapeutic agents.
